# Efficient cross-validation traversals in feature subset selection

**DOI:** 10.1038/s41598-022-25942-4

**Published:** 2022-12-12

**Authors:** Ludwig Lausser, Robin Szekely, Florian Schmid, Markus Maucher, Hans A. Kestler

**Affiliations:** 1 Institute of Medical Systems Biology, Ulm University, Ulm, Germany; 2 Faculty of Computer Science, Technische Hochschule Ingolstadt, Ingolstadt, Germany

**Keywords:** Computational science, Computer science, Scientific data

## Abstract

Sparse and robust classification models have the potential for revealing common predictive patterns that not only allow for categorizing objects into classes but also for generating mechanistic hypotheses. Identifying a small and informative subset of features is their main ingredient. However, the exponential search space of feature subsets and the heuristic nature of selection algorithms limit the coverage of these analyses, even for low-dimensional datasets. We present methods for reducing the computational complexity of feature selection criteria allowing for higher efficiency and coverage of screenings. We achieve this by reducing the preparation costs of high-dimensional subsets ${\mathscr {O}}({n}m^2)$ to those of one-dimensional ones ${\mathscr {O}}(m^2)$. Our methods are based on a tight interaction between a parallelizable cross-validation traversal strategy and distance-based classification algorithms and can be used with any product distance or kernel. We evaluate the traversal strategy exemplarily in exhaustive feature subset selection experiments (perfect coverage). Its runtime, fitness landscape, and predictive performance are analyzed on publicly available datasets. Even in low-dimensional settings, we achieve approximately a 15-fold increase in exhaustively generating distance matrices for feature combinations bringing a new level of evaluations into reach.

## Introduction

Feature subset selection (FSS) is one of the primary ingredients for constructing sparse and robust classification and clustering algorithms^1,2^. It facilitates the identification of critical components and the rejection of distracting measurements^3^. It is, therefore, not only a countermeasure for the curse of dimensionality^4^ but also a valuable tool for increasing interpretability^5^. The second one is mandatory in the scientific context, where findings on the background of a subject of interest are required for hypothesis generation. An example might be the analysis of molecular profiles in medical applications, where identifying a predictive subset of features (e.g., molecular concentrations) leads to new hypotheses on the causes or mechanisms of a disease or even reveals new drug targets^6–8^.

The requirements for FSS in the setting described above are quite different from those of other application fields. Samples are typically rare due to ethical or financial reasons. Datasets are, therefore, of a low cardinality ($m \rightarrow 0$). Often only a few dozens of samples are available, and analyses must be supplemented by foreign entities^9–11^. Although feature profiles can, in general, be very high-dimensional ($n \gg m$), the identification of informative feature subsets is also of interest for low-dimensional settings ($n \rightarrow 0$). For example, one might screen preselected panels of molecular profiles for feature subsets relevant to a specific type of disease^12–16^.

However, most FSS algorithms are not designed for in-depth analysis of a high dimensional feature space ($n \gg m$), due to its exponential increase with dimensionality^1^. They are therefore not designed for high coverage of this feature space and utilize heuristic or stochastic strategies that only evaluate a small fraction of all possible feature combinations^17^. Here, optimal feature subset selection can not be guaranteed. Higher or perfect coverage is in the range only for low dimensions ($n \rightarrow 0$) due to the lower number of features.

Rapid FSS criteria have an excellent potential for improving the coverage of these screening procedures as they allow for evaluating more feature combinations than slower ones. They, therefore, provide a more detailed outline of the feature space and a deeper insight into the influence of individual features. Nevertheless, this acceleration can come at a price as fast FSS criteria often neglect important robustness aspects, often validated in re- or subsampling experiments^5,18–20^. Alternatively, improved runtime or time complexity can also be achieved by using specialized data structures, which come at the price of an increased space complexity^21,22^. A suitable tradeoff between both resources is required^23^. However, most of these data structures are designed for screening large data collections ($n \ll m$) and lose their benefit in high dimensions ($n \gg m$)^22^. Examples can be k-d trees^24^, VP trees^25^, or BK trees^26^.

FSS algorithms are typically categorized according to their interaction with the overall classification model^27,28^. One of these categories, filter methods, operate without knowledge of the classifiers^29^. They are independent preprocessing units and often optimize autonomous criteria (e.g., correlation to class label^30^). Other FSS algorithms interact with the optimization process of the classification algorithm. Wrapper methods search for feature combinations that optimize the performance measure (e.g., accuracy) for evaluating the trained classifier^31^. They are based on suboptimal search strategies, such as the sequential forward selection^32^ or backward elimination^33^. FSS algorithms can also be embedded in a classification model’s training process to access the classifier’s inherent properties. In this case, the learning procedure is designed to construct a sparse classification model that evaluates only a small subset of features. Methods of this type are frequently used for training tree- or ensemble-classifiers^34,35^. For example, more complex or hybrid types of interactions can be found in the context of multi-class classifier systems^9,10^. FSS algorithms can also be linked to external domain knowledge to guide the selection process^8,36,37^.

Although most FSS strategies are suboptimal due to their heuristic or stochastic nature, some approaches exist that are guaranteed to find a globally optimal combination. Nevertheless, the corresponding objective functions have to fulfill some theoretical properties that are rarely applicable. Prominent examples of these FSS methods are based on branch and bound algorithms^38^, dynamic programming^4^, or greedy strategies^21^. For the general case, it has been shown that there exists no sequential non-exhaustive feature subset selection procedure that is optimal^39^.

This work presents an efficient data structure for dimensionality-invariant feature subset evaluation. That is, the costs for evaluating high-dimensional feature subsets ${\mathscr {O}}(nm^2)$ are reduced to those of one-dimensional ones ${\mathscr {O}}(m^2)$. Furthermore, we utilize this data structure for fast and efficient algorithms for re- or subsampling-based selection criteria. Both techniques can be coupled to any heuristic or stochastic FSS-strategy that operates on a Hasse diagram.

The rest of this article is organized as follows: “Methods” section introduces two proposed techniques for improving the runtime complexity of FSS strategies for distance-based classification models. We exemplarily aggregate those techniques into a combined FSS strategy, utilizing a *k*-Nearest Neighbor Classifier (e-*k*-NN) (“The exhaustive *k*-NN approach (e-*k*-NN)” section). The first technique focuses on the fast enumeration of distance matrices. The structure of distance-based classifiers allows for a quick evaluation of feature signature sequences, enabling increased search space coverage (“Distance based FSS” section). Using efficient enumeration schemes and memoization techniques allows for dimensionality-invariant costs for each feature subset. Our FSS strategy can even be applied for exhaustive subset evaluations of all feature combinations (“Exhaustive enumeration scheme” section). In this case, we cannot only identify the global optimum of a selection criterion but also reconstruct the exact fitness landscape of all feature combinations. Moreover, one can parallelize their generation into independent tasks (“Parallelization” section). The second technique addresses the relatively high runtime complexity of resampling experiments, which are required for the robust evaluation of feature combinations. We propose a fast evaluation technique for cross-validation experiments (CV) of the well-known *k*-Nearest Neighbor Classifiers (*k*-NN)^40^. The reduced complexity of these experiments extends the range and coverage of FSS strategies (“Estimation of k-nearest neighbor cross-validation complexity” section). Furthermore, it also allows the formulation of a new bound on the cross-validation error of *k*-NNs (“Cross-validation error bound” section).

We evaluate both techniques and the final e-*k*-NN strategy in $10\times 10$ cross-validation experiments on publicly available datasets (“Experiments” section). Its runtime and performance are assessed in exhaustive screens with up to $10^{12}$ feature combinations (“Results” section). Finally, a discussion of the FSS strategy and the corresponding results are given in “Discussion and conclusion” section.

## Methods

We analyze FSS in classification^41^. A classifier is a function $c: {\mathscr {X}} \rightarrow {\mathscr {Y}}$ for predicting the class label $y\in {\mathscr {Y}}$ of a given object represented by a vector of features or measurements $(x^{(1)}, \ldots , x^{(n)})^T={\textbf{x}} \in {\mathscr {X}}$. For simplicity we assume the feature space to be embedded in a *n*-dimensional real-valued space ${\mathscr {X}} \subseteq {\mathbb {R}}^n$. However, the findings presented in this manuscript also hold for heterogene data representations, where each $x^{(i)}$ has a different scale (e.g. qualitative or quantitative) or complex data type (e.g. tensors, graphs, strings, $\ldots$).

A classifier is initially adapted via a set of labeled training examples ${\mathscr {T}} = \{({\textbf{x}}_{j}, y_{j})\}_{j=1}^{m}$ and subsequently tested on an independent set of labeled validation examples ${\mathscr {V}}$, ${\mathscr {T}} \cap {\mathscr {V}}= \emptyset$. We will use the notion $c_{{\mathscr {T}}}$ if the classifier *c* is trained on the training set ${\mathscr {T}}$. The set of all available samples will be denoted by ${\mathscr {S}} = {\mathscr {T}} \cup {\mathscr {V}}$.

**Figure 1:**
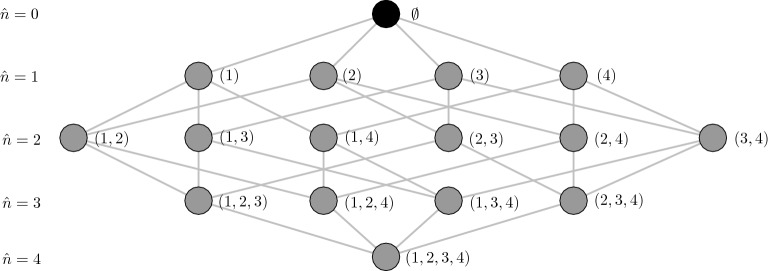
FSS search space: The figure provides the FSS search space ${\mathscr {I}}$ for $n=4$ features. It comprises $2^n-1$ non-empty feature subsets (gray nodes) from which one has to be selected. The empty set $\emptyset$ (black node) is typically omitted. The feature subsets are organized row-wise according to their feature set size ${\hat{n}}$. The FSS search space is shown in form of a Hasse diagram. That is two nodes are connected via an edge if both of them can be translated into the other by adding or removing a feature.

FSS is an optional subprocess within the training of a classifier. Its task is the selection of a suitable feature combination from the set of $2^n-1$ possible ones. Deselected features can no longer influence the prediction of the classifier. The underlying search space of a FSS problem is illustrated Hasse diagram as shown in Fig. 1. A selected feature set of size ${\hat{n}}$ will be described by a sorted and repetition free index vector ${\textbf{i}} = \left( i_{1}, \ldots , i_{{\hat{n}}}\right) ^{T}$,

1 $$\begin{aligned} {\textbf{i}} \in {\mathscr {I}} = \{{\textbf{i}} \in {\mathbb {N}}^{{\hat{n}} \le n} \, | \, i_{k-1} < i_{k}, \, 1 \le i_{k} \le n\}. \end{aligned}$$

The corresponding reduced representation of a sample ${\textbf{x}}\in {\mathbb {R}}^{n}$ will be given by ${\textbf{x}}^{({\textbf{i}})} = \left( x^{(i_1)}, \ldots , x^{(i_{{\hat{n}}})} \right) ^T$. The notation $\hat{{\textbf{i}}} = \{i_{j}\}_{j=1}^{{\hat{n}}}$ will be used to denote the unordered set of indices.

The generalization ability of a classifier can be evaluated in a $r \times f$ cross-validation (CV) experiment^41^, where *r* denotes the number of runs and *f* the number of folds. This subsampling experiment is a summary of *r* independent runs of a *f*-fold CV experiment. A single *f*-fold CV divides the overall set of samples ${\mathscr {S}}$ into *f* folds of approximately equal size. In an iterative procedure, each of the folds is used as a validation set while the remaining folds are used for training the classifier. The mean error rate over all folds and runs is afterwards used as an estimate of the classifier’s generalization error

2 $$\begin{aligned} {\hat{R}}_{cv}({\mathscr {S}}) = \frac{1}{r|{\mathscr {S}}|}\sum _{i=1}^{r}\sum _{j=1}^{f} \sum _{({\textbf{x}},y) \in {\mathscr {V}}_{ij}} {\mathbb {I}}_{\left[ c_{{\mathscr {T}}_{ij}} ({\textbf{x}}) \ne y \right] }. \end{aligned}$$

A special case is the $1 \times |{\mathscr {S}}|$ CV which is also called leave-one-out cross-validation (LO). Here, each sample is evaluated separately. The symbol ${\hat{R}}_{lo}$ is used to denote the corresponding error estimate.

### The exhaustive *k*-NN approach (e-*k*-NN)

In the following, we propose two techniques for improving the runtime complexity of distance-based feature selection criteria. The first one is an efficient memoization and enumeration scheme for the calculation of distance matrices (“Distance based FSS” and “Exhaustive enumeration scheme” sections). The corresponding sequence of feature sets can be split into an arbitrary number of junks allowing an easy parallelization with individual workload for each compute note (“Parallelization” section). The second one is a fast evaluation technique for CV experiments based on *k*-NNs (“Nearest neighbor classification” and “Estimation of k-nearest neighbor cross-validation complexity” sections). It implies a new error bound for the CV error of *k*-NNs (“Cross-validation error bound” section).

We demonstrate the performance of these techniques in exhaustive FSS experiments. That is, the chosen selection criterion is evaluated for each of the $2^n-1$ feature subsets. In this case, the selection strategy is free from subsampling effects as induced by stochastic or heuristic strategies. It is the only selection strategy that is guaranteed to find a global optimum solution for any type of objective^42,43^. As a selection criterion, the minimal $r \times f$ CV error of a *k*-NN was chosen

3 $$\begin{aligned} \underset{{\textbf{i}}\in {\mathscr {I}}}{\mathrm {arg\,min}} \quad {\hat{R}}_{cv}({\mathscr {T}}_{{\textbf{i}}}). \end{aligned}$$

Note that the evaluation of ${\textbf{i}}$ is performed on the training set of a classifier, where ${\mathscr {T}}_{{\textbf{i}}} = \{({\textbf{x}}^{({\textbf{i}})},y) \mid ({\textbf{x}},y) \in {\mathscr {T}} \}$ denotes the training set’s restriction to the measurements in ${\textbf{i}}$. The selection process is therefore based on an internal CV. We finally evaluated this overall strategy as exhaustively feature selecting *k*-NN classifiers (*e*-*k*-NN) in (“Results” section.

### Distance based FSS

The optimization criterion given in Eq. (3) can be seen as a distance based FSS criterion. That is, the assessment of a feature subset is based on pairwise distances *d*(., .) measured on the training samples, where

4 $$\begin{aligned} d: {\mathscr {X}} \times {\mathscr {X}} \rightarrow {\mathbb {R}}^{+}_{0} \end{aligned}$$

These measurements are summarized in a distance matrix ${\textbf{D}}$:

5 $$\begin{aligned} {\textbf{D}}_{{\textbf{i}}} := \left( \begin{matrix} d\left( {\textbf{x}}_{1}^{({\textbf{i}})},{\textbf{x}}_{1}^{({\textbf{i}})} \right) &{} &{} d\left( {\textbf{x}}_{1}^{({\textbf{i}})},{\textbf{x}}_{m}^{({\textbf{i}})} \right) \\ &{}\ddots &{}\\ d\left( {\textbf{x}}_{m}^{({\textbf{i}})},{\textbf{x}}_{1}^{({\textbf{i}})} \right) &{} &{} d\left( {\textbf{x}}_{m}^{({\textbf{i}})},{\textbf{x}}_{m}^{({\textbf{i}})} \right) \\ \end{matrix}\right) . \end{aligned}$$

Index ${\textbf{i}}$ will be used to indicate the selected feature subset.

In the following, we assume that this distance measure is decomposable. That is, it can be seen as sum of the feature-wise (one dimensional) distances:

6 $$\begin{aligned} {\textbf{D}}_{{\textbf{i}}} = \sum _{i \in \hat{{\textbf{i}}}} {\textbf{D}}_{(i)} \,. \end{aligned}$$

Distance measures that fulfill this criterion are for instance the (potentiated) Minkowski metrices or product distances of type $d(.,.) = \sum _{i \in \hat{{\textbf{i}}}} d_{i}(.,.)$, which evaluate each feature by a separate distance^44^ or kernel^45^. We will utilize a variant of the Euclidean distance (no square root) as a canonical example

7 $$\begin{aligned} d^{2}_{2}({\textbf{x}}_{s}, {\textbf{x}}_{t}) = \sum ^{n}_{i=1} \left( x_{s}^{(i)} - x_{t}^{(i)} \right) ^2 \quad . \end{aligned}$$

A standard implementation for calculating this distance matrix in an $|\hat{{\textbf{i}}}|$ dimensional space can be done in time ${\mathscr {O}}(m^2|\hat{{\textbf{i}}}|)$.

As a distance-based FSS algorithm has to calculate a distance matrix for each feature subset evaluation a reduction of this computational complexity is desirable and extends the coverage of the search space of feature subsets ${\mathscr {I}}$. For a decomposable distance the complexity can be improved by memorizing distance matrices. For example, if the distance matrices ${\textbf{D}}_{{\textbf{i}}'}$ and ${\textbf{D}}_{{\textbf{i}}''}$ are known (e.g. from previous evaluations), where $\hat{{\textbf{i}}}' \cap \hat{{\textbf{i}}}'' = \emptyset$ and $\hat{{\textbf{i}}}' \cup \hat{{\textbf{i}}}'' = \hat{{\textbf{i}}}$, the distance matrix ${\textbf{D}}_{{\textbf{i}}}$ can be calculated via a single matrix addition ${\textbf{D}}_{{\textbf{i}}'}+ {\textbf{D}}_{{\textbf{i}}''}$. This reduces the computational complexity to ${\mathscr {O}}(m^2)$.

Especially FFS algorithms that modify existing distance matrices by adding or removing individual features can benefit from this effect by memorizing all *n* feature-wise distance matrices ${\textbf{D}}_{(i)}$. In this case, each modification can be seen as a single matrix addition (or subtraction). This holds for all search algorithms that directly operate on the structure of the Hasse diagram. The memorization requires once an additional time and space complexity of ${\mathscr {O}}(nm^2)$. For a large number of evaluations $e\gg n$ we get a time complexity of

8 $$\begin{aligned} {\mathscr {O}}(em^2 + nm^2) = {\mathscr {O}}(em^2) \end{aligned}$$

against the naive time complexity of ${\mathscr {O}}(enm^2)$.

### Exhaustive enumeration scheme

Remarkably, this reduction can be achieved by exhaustive search algorithms, if the feature signatures are processed in lexicographical order^46^. The computational complexity is than reduced to ${\mathscr {O}}(2^n m^2)$.

#### Definition 1

Let ${\textbf{i}} = (i_1, \ldots , i_k)^{T}$ and ${\textbf{j}} = (j_1, \ldots , j_l)^{T}$ denote sorted and repetition free index vectors ${\textbf{i}}, {\textbf{j}} \in {\mathscr {I}}$. The index vector ${\textbf{i}}$ is said to be *lexicographically smaller* than ${\textbf{j}}$ denoted by ${\textbf{i}} \sqsubset {\textbf{j}}$ if and only if

9 $$\begin{aligned} k < l:&\forall s \le k:&i_{s} = j_{s} \quad \quad \quad \quad \quad \quad \text {or} \end{aligned}$$

10 $$\begin{aligned} \exists t< m:&\forall s \le t:&i_{s} = j_{s} \wedge i_{t+1} < j_{t+1}, \end{aligned}$$

where $m = \min {(k,l)}$. Vector ${\textbf{i}}$ is called *parent* of ${\textbf{j}}$, if ${\textbf{i}} \sqsubset {\textbf{j}}$ and $k=l-1$.

Assuming that all feature-wise distance matrices ${\textbf{D}}_{(i)}$ are already memorized for all *n* one-dimensional subspaces $i \in \{1, \ldots , n\}$, the distance matrices for any multi dimensional index vector ${\textbf{i}} = (i_1, \ldots , i_k)^{T}$, can be calculated by a single matrix addition:

11 $$\begin{aligned} {\textbf{D}}_{{\textbf{i}}}={\textbf{D}}_{{\textbf{i}}'} + {\textbf{D}}_{(i_{k})} \quad . \end{aligned}$$

**Figure 2:**
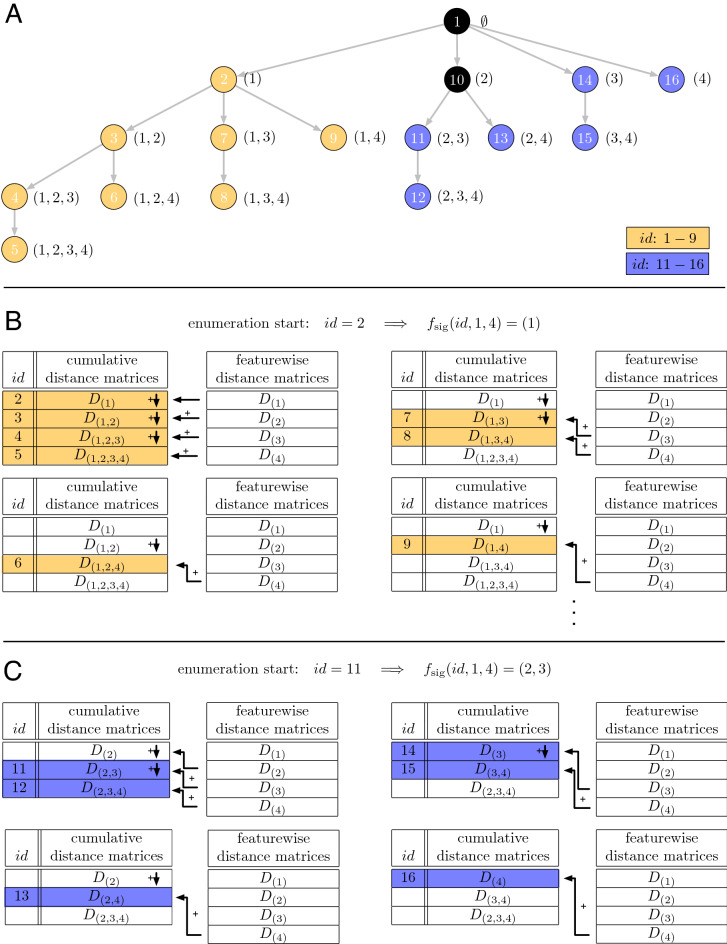
Enumeration scheme for all feature combinations. (**A**) shows the tree, structuring all index vectors for $n=4$ features according to the parent relationship. Filled circle numbers give the sequence of evaluation, numbers in parentheses show the chosen feature numbers. (**B**) and (**C**) give examples for the in-place update of the cumulative distance matrices. The memorized cumulative and feature-wise distance matrices are shown. The arrows indicate the distance matrices that are utilized for calculating the cumulative distance matrix with index *id*. The lookup structure consists of at most 2*n* distance matrices. The example of panel B starts the updates from the beginning of the sequence ($id=2$). The example of panel C is started by an arbitrarily chosen feature set index ($id = 11$).

Here ${\textbf{i}}'$ denotes the parent of ${\textbf{i}}$. Structuring the set of all possible feature combinations according to the parental relationship leads to the construction of a search tree (Fig. 2A). Here it can be observed that at most *n* additional parental nodes have to be memorized (Fig. 2B). Overall at most 2*n* matrices are required, which corresponds to a space complexity of ${\mathscr {O}}(nm^2)$.

### Parallelization

In order to facilitate parallelization, the procedure described above can be modified to start at an arbitrary index vector ${\textbf{i}}$ (Fig. 2C). Memorizing the distance matrices for the sequence of parental index vectors of ${\textbf{i}}$ and the feature-wise matrices allows calculating the remaining distance matrices ${\textbf{D}}_{{\textbf{j}}}$, ${\textbf{i}} \sqsubset {\textbf{j}}$ as mentioned before. In this way, the complete sequence of all feature signatures can be split into partial sequences of arbitrary length and can afterwards be processed in parallel. The length of the partial sequences might be chosen in order to optimize the load balancing of the available compute cores^47^. For each parallel call, a copy of the feature-wise and cumulative distance matrices are required.

The starting points for the partial sequences can be addressed directly without enumerating predecessor signatures. An example is given in Table 1. A feature signature ${\textbf{i}}$ can be identified and reconstructed from its position *id* in the lexicographical order and the total number of features *n*. The corresponding mapping will be called $f_{sig}(id,n)=f_{sig}(id,n,1)$ in the following

12 $$\begin{aligned} f_{sig}(id, i, n) = {\left\{ \begin{array}{ll} \emptyset &{} \quad \text {if } id\le 1 \\ f_{sig}(id - 2^{n-i}, i+1, n) &{} \quad \text {if } id \ge 2^{n-i}+1\\ (i,j_1, \ldots , j_l)&{} \quad \text {otherwise} \end{array}\right. }, \end{aligned}$$

where $(j_1, \ldots , j_l) = f_{sig}(id-1, i+1, n)$. The recursive formula $f_{sig}(id, i, n)$ will stepwise elongate the partial signature until all required feature indices are incorporated. In this context variable *i* can be seen as the index of the *i*th feature. It is initialized with $i=1$. Starting with the first element, $f_{sig}$ will traverse stage-wise through the search tree of the enumeration scheme. The current position of the feature signature is filled with *i*, if $id \in \left[ 2, 2^{n-i} \right]$. If $id > 2^{n-i}$, the search is continued with feature index $i:=i+1$ and a diminished $id := id - 2^{n-i}$. If the current position is filled, a recursive call of $f_{sig}$ fills the next position of the signature. This screen takes place on the next stage of the search tree. As we operate on sorted and repetition free index vectors, the next possible feature index is $i:=i+1$.

The position *id* of a signature ${\textbf{i}}$ can also be calculated directly, if the total number of features *n* is known. The corresponding function will be denoted as

13 $$\begin{aligned} f_{id}({\textbf{i}},n) = 1+\sum _{1 \le p \le |{\textbf{i}}|} \left( 1+ \sum _{i_{p-1}< i < i_{p}} 2^{(n-i)} \right) . \end{aligned}$$

Here we assume that $| \emptyset | = 0$, $i_{0} = 0$ and $i,p \in {\mathbb {N}}$.

**Table 1:** Direct mapping of signature indices *id* and feature signatures ${\textbf{i}}$. The table lists an example for $n=4$ features according to the proposed enumeration scheme. The *id* of a feature signature ${\textbf{i}}$ can be calculated directly via the function call $f_{id}({\textbf{i}}, n)$. The feature signature of an *id* is given by ${\textbf{i}}=f_{sig}(id,i=1,n=4)$.

Index *id*	Feature signature ${\textbf{i}}$
1	$\emptyset$
2	$(1)^T$
3	$(1,2)^T$
4	$(1,2,3)^T$
5	$(1,2,3,4)^T$
6	$(1,2,4)^T$
7	$(1,3)^T$
8	$(1,3,4)^T$
9	$(1,4)^T$
10	$(2)^T$
11	$(2,3)^T$
12	$(2,3,4)^T$
13	$(2,4)^T$
14	$(3)^T$
15	$(3,4)^T$
16	$(4)^T$
$id = f_{id}({\textbf{i}}, n)$.	${\textbf{i}}=f_{sig}(id,i=1,n=4)$

### Nearest neighbor classification

In the following, we concentrate on Nearest Neighbor Classifiers (*k*-NN)^40^ as a conceptually simple example for a distance-based classifier. The *k*-NNs are prototype-based classifiers that utilize all available training samples as prototypes (${\mathscr {P}} = {\mathscr {T}}$). They predict the class of a new unseen query point ${\textbf{v}} \in {\mathbb {R}}^{n}$ by a majority vote on the class labels of the *k* nearest neighbors in ${\mathscr {P}}$

14 $$\begin{aligned} c({\textbf{v}}) = \underset{{y\in {\mathscr {Y}}}}{\textrm{arg max}} \, \bigl | \left\{ ({\textbf{x}},y) \in \textrm{NN}_{k}({\textbf{v}}, {\mathscr {P}}) \right\} \bigr |. \end{aligned}$$

Here, $\textrm{NN}_{k}({\textbf{v}}, {\mathscr {P}})$ denotes the *k* neighborhood of ${\textbf{v}}$ in ${\mathscr {P}}$,

15 $$\begin{aligned} \textrm{NN}_{k}({\textbf{v}}, {\mathscr {P}}) = \Bigl \{ ({\textbf{x}},y) \in {\mathscr {P}} \,|\, \textrm{rk}_{D_{{\textbf{v}}}} \bigl (d({\textbf{v}}, {\textbf{x}})\bigr )\le k \Bigr \} \end{aligned}$$

and $\textrm{rk}_{D_{{\textbf{v}}}}$ the rank function over pairwise distances *d*(., .) between ${\textbf{v}}$ and the members of ${\mathscr {P}}$

16 $$\begin{aligned} D_{{\textbf{v}}} = \bigl \{ d({\textbf{v}}, {\textbf{x}}) \,|\, ({\textbf{x}}, y) \in {\mathscr {P}}\bigr \}. \end{aligned}$$

The *k*-NN is therefore parameterized by the chosen neighborhood size *k* and the chosen distance measure *d*.

The computational complexity of applying *k*-NN corresponds to the complexity of finding *k* times the minimum distance between the training samples and the query sample ${\mathscr {O}}(k|\hat{{\textbf{i}}}||{\mathscr {T}}|)$. The factor $|\hat{{\textbf{i}}}|$ can again be removed, if the *k*-NN is embedded in an exhaustive FSS experiment.

### Estimation of k-nearest neighbor cross-validation complexity

The computational costs for a $r \times f$ CV evaluation of the *k*-NN can be reduced by memorizing different aspects of the global data set ${\mathscr {S}}$ of all available samples. The number of distance calculations

17 $$\begin{aligned} rf |{\mathscr {V}}_{st}| \cdot k |{\mathscr {T}}_{st}| = rf\underbrace{\frac{1}{f} |{\mathscr {S}}|}_{=|{\mathscr {V}}_{st}|} \cdot k \underbrace{\frac{f-1}{f} |{\mathscr {S}}|}_{= |{\mathscr {T}}_{st}|} = rk \frac{f-1}{f}|{\mathscr {S}}|^2 \end{aligned}$$

can be reduced to $|{\mathscr {S}}|^2$ by precalculating the global distance matrix. Here, ${\mathscr {T}}_{st}$ and ${\mathscr {V}}_{st}$ denote the training and test sets of a single experiment. We assume that $|{\mathscr {S}}|$ is dividable by *f*.

**Figure 3:**
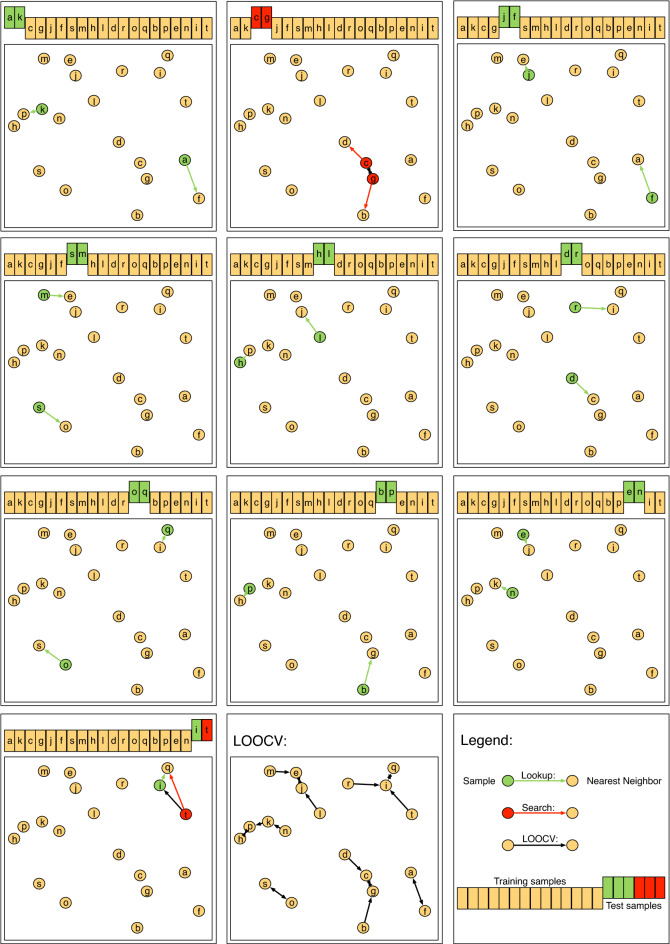
Fast cross validation of a *k*-NN: The figure shows the relationship of a leave-one-out cross validation (LOOCV) and a *f*-fold cross validation ($f=10$, 10-fold CV) for the example of a 1-NN classifier. A dataset of twenty samples is analysed (circles). The center panel at the bottom shows the resulting neighborhood graph of the LOOCV. Each sample is connected with its global nearest neighbor (sample $\rightarrow$ neighbor). The other panels show the individual classification experiments of the 10-fold CV. For each experiment, only the training samples and the current test sample are available. If the global nearest neighbor of the test sample is included in the training set, the search for the nearest neighbor can be replaced by a look up (green). Otherwise, the training set has to be screened (red).

The number of distance comparisons can be reduced via a related strategy (Fig. 3). For a given test sample ${\textbf{v}} \in {\mathscr {V}}_{st}$, it is likely that there is an overlap between its *k* nearest neighbors in the current training set ${\mathscr {T}}_{st}$ and the *k* nearest neighbors among the samples in ${\mathscr {S}}$. The second one will be called global nearest neighbors in the following. If one of the global nearest neighbors is included in the current training set the corresponding search ($|{\mathscr {T}}_{st}|$ distance comparisons) can be replaced by a single lookup. If the current *k* nearest neighbors correspond to the *k* global nearest neighbors the prediction of ${\textbf{v}}$ is equal for the current training/test split and LO. The occurrence of a successful lookup *lu* is distributed according to a hypergeometric distribution $lu \sim HG(k, |{\mathscr {S}}|-1, |{\mathscr {T}}|)$. In expectation

18 $$\begin{aligned} {\mathbb {E}}\left[ lu\right] = k \frac{|{\mathscr {T}}|}{|{\mathscr {S}}|-1} = k \frac{f-1}{f} \frac{|{\mathscr {S}}|}{|{\mathscr {S}}|-1} \end{aligned}$$

nearest neighbors can be found for each test sample.

For the $|{\mathscr {V}}|$ validation samples in each of the $r \times f$ in each folds, the following number unsuccessful lookups is expected

19 $$\begin{aligned} rf \left( k|{\mathscr {V}}| - {\mathbb {E}}\left[ lu\right] |{\mathscr {V}}|\right)&= rf|{\mathscr {V}}| \left( k- {\mathbb {E}}\left[ lu\right] \right) \end{aligned}$$

20 $$\begin{aligned}&= rf\frac{1}{f}|{\mathscr {S}}| \left( k- k \frac{f-1}{f} \frac{|{\mathscr {S}}|}{|{\mathscr {S}}|-1}\right) \end{aligned}$$

21 $$\begin{aligned}&= rk|{\mathscr {S}}| \left( 1- \frac{f-1}{f} \underbrace{\frac{|{\mathscr {S}}|}{|{\mathscr {S}}|-1} }_{\ge 1}\right) \end{aligned}$$

22 $$\begin{aligned}&\le rk|{\mathscr {S}}|\left( 1 - \left( \frac{f-1}{f}\right) \right) \end{aligned}$$

23 $$\begin{aligned}&= rk\left( \frac{1}{f}\right) |{\mathscr {S}}|. \end{aligned}$$

This reduces the number of required distance comparisons to at least

24 $$\begin{aligned} rk\left( \frac{1}{f}\right) |{\mathscr {S}}||{\mathscr {T}}| = rk\left( \frac{f-1}{f^2}\right) |{\mathscr {S}}|^2. \end{aligned}$$

In contrast to the distance calculations, the lookup table for the distance comparisons must be calculated explicitly. The costs for this lookup table are approximately given by $k|{\mathscr {S}}|^2$ distance comparisons. The overall time complexity of a single *k*-nearest neighbor experiment summarizes then to

25 $$\begin{aligned} {\mathscr {O}}\left( rk \frac{f-1}{f^2}|{\mathscr {S}}|^2 + (k+1)|{\mathscr {S}}|^2 \right) . \end{aligned}$$

The space complexity of the additional lookup structure is ${\mathscr {O}}\left( k|{\mathscr {S}}|\right)$.

### Cross-validation error bound

Runtime optimisation via lookups can also be used to compute lower and upper bounds on the cross-validation error ${\hat{R}}_{cv}$ of *k*-NN. This bound depends on the classifier’s leave-one-out error and the number of folds *f*.

Let ${\hat{R}}_{cv} = {\hat{R}}_{cv}({\mathscr {S}})$ and ${\hat{R}}_{lo} = {\hat{R}}_{lo}({\mathscr {S}})$ denote the risk estimate of a *f*-fold cross-validation and a leave-one-out cross-validation respectively. Given a random split of an *f*-fold cross-validation, the global *k* nearest neighbors of any data point are also included in the current training set ${\mathscr {T}}$ with probability

26 $$\begin{aligned} p_{lo}&= \frac{\left( {\begin{array}{c}|{\mathscr {T}}|\\ k\end{array}}\right) \left( {\begin{array}{c}|{\mathscr {V}}|-1\\ 0\end{array}}\right) }{\left( {\begin{array}{c}|{\mathscr {S}}|-1\\ k\end{array}}\right) } = \frac{|{\mathscr {T}}|! (|{\mathscr {S}}|-1-k)!}{(|{\mathscr {S}}|-1)!(|{\mathscr {T}}|-k)!} \end{aligned}$$

27 $$\begin{aligned}&=\frac{\prod _{i=|{\mathscr {T}}|-k+1}^{|{\mathscr {S}}|-1-k}i}{\prod _{i=|{\mathscr {T}}|+1}^{|{\mathscr {S}}|-1}i} =\frac{\prod _{i=|{\mathscr {T}}|+1}^{|{\mathscr {S}}|-1}(i-k)}{\prod _{i=|{\mathscr {T}}|+1}^{|{\mathscr {S}}|-1}i} \end{aligned}$$

28 $$\begin{aligned}&=\prod _{i=|{\mathscr {T}}|+1}^{|{\mathscr {S}}|-1}\left( 1-\frac{k}{i}\right) . \end{aligned}$$

Examples are shown in Table 2.

**Table 2:** Examples for probability $p_{lo}$ (in %) in dependence on the number of considered neighbors *k* and the dataset size $|{\mathscr {S}}|$. The examples are based on 10-fold CV experiments.

*k*	$|{\mathscr {S}}|$					
	50	60	70	80	90	100
1	91.8	91.5	91.3	91.1	91.0	90.9
3	77.0	76.3	75.8	75.4	75.1	74.9
5	64.1	63.2	62.5	62.1	61.7	61.4
7	52.8	51.9	51.3	50.8	50.5	50.2

With probability $p_{lo}$, CV and LO lead to the same classification of a data point (cf. Eq. 18). For those cases in which the global *k* nearest neighbor is not included in the training set, let $R_u$ denote the (unknown) error rate of the resulting predictions. Now the expected CV error can be written as

29 $$\begin{aligned} {\mathbb {E}}_{F}\left[ {\hat{R}}_{cv}\right] = p_{lo} {\hat{R}}_{lo} + (1-p_{lo}) {\mathbb {E}}_{F}\left[ R_{u}\right] , \end{aligned}$$

where ${\mathbb {E}}_{F}$ denotes the expectation value under the uniform distribution *F* of all possible splits into *f* folds. Since $0 \le {\mathbb {E}}_{F}\left[ R_{u}\right] \le 1$,

30 $$\begin{aligned} p_{lo} {\hat{R}}_{lo} \le {\mathbb {E}}_{F}\left[ {\hat{R}}_{cv}\right] \le p_{lo} {\hat{R}}_{lo} + (1-p_{lo}). \end{aligned}$$

This can be rewritten as

31 $$\begin{aligned} p_{lo} {\hat{R}}_{lo} \le {\mathbb {E}}_{F}\left[ {\hat{R}}_{cv}\right] \le 1+p_{lo}\left( {\hat{R}}_{lo}-1\right) . \end{aligned}$$

## Experiments

Our experiments focus on three different aspects of the exhaustive FSS. Its runtime (“Runtime experiments” section), fitness landscape (“Exhaustive screening experiments” section) and generalization ability (“Cross-validation experiments” section) are evaluated. In all experiments, the *e*-*k*-NN is validated for $k \in \{1,3,5,7\}$. Real-world datasets were downloaded from the UCI machine learning repository^48^ (Table 3). Their number of samples lies in the range of $m=[166;2126]$ and their number of features in the range of $n=[9;22]$.

**Table 3:** datasets analyzed in $\mathbf {10\times 10}$ CV experiments.

Nr.	name	Number of classes ($|{\mathscr {Y}}|$)	Number of features (*n*)	Number of samples (*m*)	Number of samples per cl. ($m_{y}$)
$d_{1}$	Heart failure^49^	2	12	299	203 + 96
$d_{2}$	Wine^50^	3	13	178	59 + 71 + 48
$d_{3}$	Statlog (Aust.)^51^	2	14	690	307 + 383
$d_{4}$	Breast Cancer Coimbra^52^	2	9	116	52 + 64
$d_{5}$	Parkinsons^53^	2	22	195	48 + 147
$d_{6}$	Segmentation^48^	6	19	1980	6 $\times$ 330
$d_{7}$	Cardiotocography^54^	3	21	2126	1655 + 295 + 176
$d_{8}$	Accent recognition^55^	6	12	329	29 + 30 + 30 + 30 + 45 + 165

### Runtime experiments

Runtime experiments were performed on an Intel(R) Xeon(R) Platinum Processor 8168 with 2.7 GHz (100 cores with HT) and 96 GB RAM. The runtime of both the exhaustive enumeration scheme and the corresponding distance-based feature selection strategy (*e*-*k*-NN) is investigated in experiments on artificial datasets with $m \in \{50,100\}$ samples and $n \in \{30,\ldots ,40\}$ features. Each sample ${\textbf{x}}$ was drawn i.i.d from ${\textbf{x}}\sim {\mathscr {U}}(0,1)^n$. A maximal time limit of 14 days ($\approx 1.21 \cdot 10^9$ ms) was used.

The exhaustive enumeration scheme is compared to a de novo calculation of all distance matrices. For the runtime evaluation of the *e*-*k*-NN a $10\times 10$ CV is used as an internal validation method. It is performed with and without the use of the lookup strategy (“Estimation of k-nearest neighbor cross-validation complexity” section). All experiments are based on multi-core evaluations on $p=100$ cores.

### Exhaustive screening experiments

The exhaustive FSS presented above provides a census on the CV accuracies achieved by *k*-NN over all feature combinations. The corresponding fitness landscape can be evaluated and visualized in order to provide information on the influence of individual features and the potential success of FSS.

In an explorative analysis, we provide the (exact) distributions of accuracies for several real datasets (Table 3) in order to identify patterns that may hint at a success or failure FSS. The accuracies are organized in histograms according to their underlying signature size ${\hat{n}}$.

### Cross-validation experiments

The *e*-*k*-NN can be applied as a standalone classification algorithm, which itself can be evaluated in (outer) $10 \times 10$ CV experiments. We utilized 8 UCI datasets (Table 3) to compare the accuracy of *e*-*k*-NN to the accuracy of various reference classifiers. The standard *k*-NN ($k\in \{1,3,5,7\}$), support vector machines with the 2-norm (*L*2-SVM, cost=1) and 1-norm (*L*1-SVM, cost=1)^56^, classification trees^34^ (CART), C4.5 decision trees^57^, learning vector quantization^58^ (LVQ) and nearest centroid classifiers^59^ (NCC) were chosen. Of note, *L*1-SVM, CART, and C4.5 are embedded feature subset selection classification algorithms. As the *e*-*k*-NN is trained $10 \times 10$ on different training sets, its internal exhaustive FSS will return up to 100 feature signatures in these experiments. The mean number of features and their standard deviation will be reported.

## Results

The following section provides the results of the three experimental setups.

### Runtime

**Figure 4:**
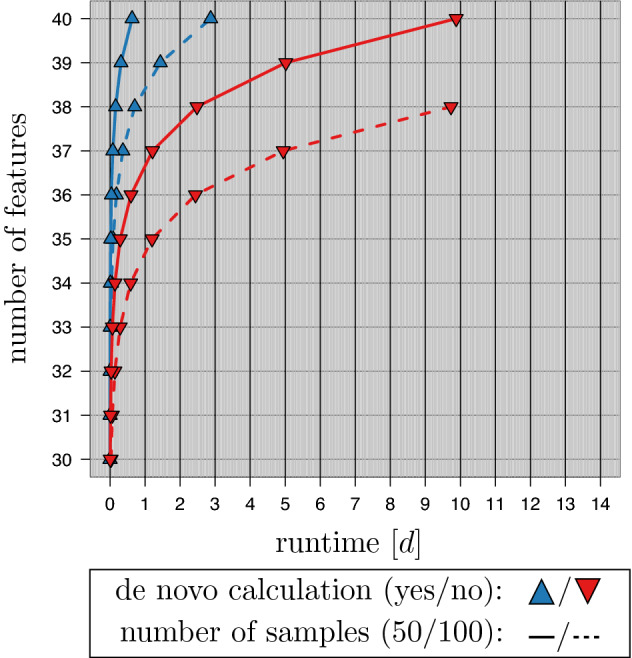
Runtime of exhaustive distance calculations. The figure summarizes runtime experiments on artificial datasets with $n \in \{30,\ldots ,40\}$ features (100 cores). In each experiment, the distance matrices of all $2^n-1$ feature combinations are generated. A maximal runtime limit of 14 days ($\approx 1.21 \cdot 10^9$ ms) was used. A comparison of a de novo calculation (lower-red triangle) and the proposed traversal strategy (upper-blue triangle) on datasets with $m \in \{50,100\}$ samples is given.

**Figure 5:**
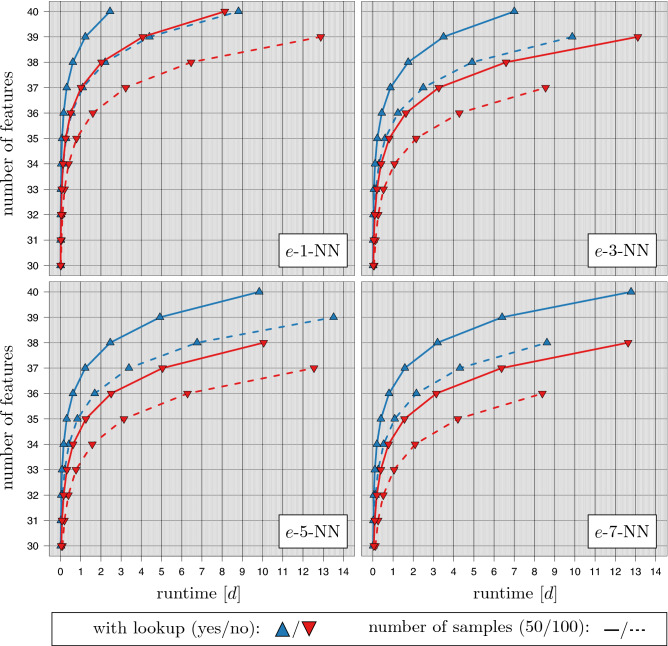
Runtime experiments with *e*-*k*-NN. The figure shows the runtime of experiments with *e*-*k*-NN ($k\in \{1,3,5,7\}$, 100 cores) on artificial datasets with $m \in \{50,100\}$ samples. Each feature combination was evaluated in a $10\times 10$ CV experiment. A maximal runtime limit of 14 days ($\approx 1.21 \cdot 10^9$ ms) was used. The runtime of *e*-*k*-NN with and without lookups (upper-blue/lower-red triangle) on artificial datasets with varying dimensionality $n \in \{30,\ldots ,40\}$ is shown.

The results of the runtime experiments are shown in Figs. 4 and 5. They are based on a parallelization of 100 cores. Figure 4 provides the results of the exhaustive distance calculations. For $m=50$ samples the (standard) de novo calculation achieved the enumeration for $n=40$ in 9d, 21h, 9min. The proposed traversal strategy required 15h 8min. For $m=100$ samples the de novo calculation was only able to handle a dimensionality of $n=38$ in the given time limit (9d, 17h, 35min). The traversal strategy was able to handle $n=40$ in 2d, 20h, 58min.

The evaluation of the *e*-*k*-NN strategy is given in Fig. 5. In this experiment, the enumeration of the distance matrices is coupled to the workload of a $10\times 10$ CV. The *e*-*k*-NN was applied with and without the use of its internal lookup strategy. As the artificial datasets were explicitly designed to provide no class information the gained speedup can be seen as a baseline for the speedup in real experiments. It lies at most by 4.13 for $m=50$ and 3.96 for $m=100$.

As expected the runtime increases for higher values of *k* in all settings. For $m=50$ samples the lookup strategy allowed for gaining all results in the chosen time limit. For $k=1$ the calculations for $n=40$ features required 2d, 10h, 49 min; for $k=7$ 12d, 18h 44 min were spent. Without the lookup strategy $n=40$ was only achieved for $k=1$ (8d, 3h, 6 min). For $k \in \{3,5\}$ at most $n=39$ were analyzed. For $k=7$ the maximum number of features was $n=38$ (12d, 15h, 13 min).

For $m=100$ samples and the use of the lookup strategy $n=40$ features were only accomplished for $k=1$ (8d, 19h, 24 min). Up to $n=39$ features were analyzed for $k \in \{3,5\}$ . Only $n=38$ features were analyzed for $k=7$ (8d, 14h, 46 min). Without the lookup strategy the runtime again increased leading to $n=39$ features for $k=1$ (12d, 21h, 7 min) down to $n=36$ for $k=7$ (8d, 9h, 20 min).

### Fitness landscape

**Figure 6:**
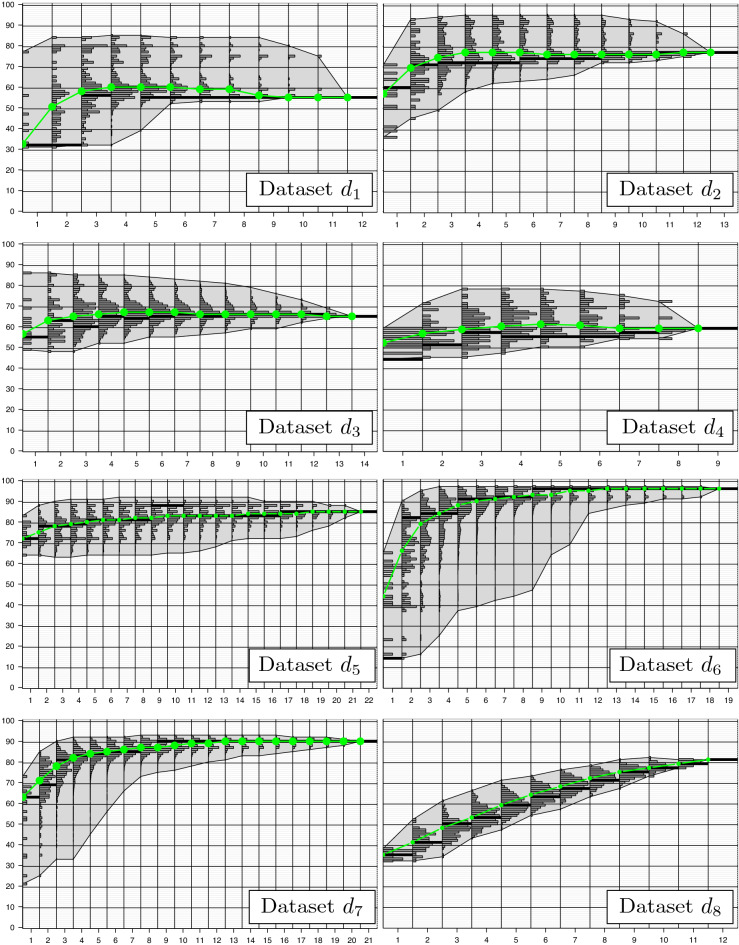
Fitness landscapes. The figure shows the fitness landscapes of e-*k*-NN, $k=1$, for datasets $d_{1} - d_{8}$. The figures for $k \in \{3,5,7\}$ can be found in the supplementary information. A fitness landscape provides the accuracies of all analyzed feature combinations. They are organized in histograms for the individual feature set sizes ${\hat{n}}$ (column-wise). The height of a histogram is normalized by the mode of the corresponding set. The underlying polygon combines the minimal and the maximal accuracies achieved for ${\hat{n}}$. For each feature combination, the mode is shown in black within the single histograms. The median of each combination is depicted in green.

In our explorative analysis, we recorded the (internal) CV accuracy of each feature combination. A summary for $k=1$ is given in Fig. 6. An overview of the remaining experiments ($k \in \{3,5,7\}$) is given in the supplementary information. All figures organize the accuracies according to the feature set sizes (*x*-axis), where each column provides a histogram of the feature combinations of size ${\hat{n}}$.

For each dataset, the leftmost histogram shows the accuracies achieved by the *n* individual features (${\hat{n}}=1$). It characterizes the performance of the features as univariate standalone markers. Over all datasets, standalone markers achieved accuracies in the range of $\left[ 14.2\%;87.2\% \right]$. For the individual datasets, the largest range of $\left[ 14.4\%;64.5\% \right]$ was observed for $d_{6}$ $(k=7)$. The smallest one of $\left[ 31.8\%;37.6\% \right]$ was found for $d_{1}$ $(k=1)$. For all experiments the multivariate feature combinations (${\hat{n}}>1$) achieve better maximal accuracies than the univariate ones. In general, the quantity increases already for small feature set sizes.

Higher ranges of accuracies are observed for multivariate feature combinations. While the ranges initially increase for smaller ${\hat{n}}$ they decrease for ${\hat{n}} \rightarrow n$. This might be caused by the underlying number of combinations $$\left( {\begin{array}{c}n\\ {\hat{n}}\end{array}}\right) $$, which is maximal for ${\hat{n}}=n/2$. Another reason might be the overlap of the corresponding feature signatures. While feature signatures can be constructed from distinct features for smaller ${\hat{n}}$, they inevitably overlap for larger ${\hat{n}}$ resulting in similar distance information.

The rightmost histogram (single bar) provides the accuracy gained by the full set of features (${\hat{n}}=n$). For each experiment, feature subsets exist that outperform the full set. This can be observed for $10.0\%$ to $100.0\%$ of the feature set sizes (mean: $84.0\%$). Feature subsets leading to the highest accuracies utilize $14.3\%$ to $91.7\%$ of all features with a mean value of $48.2\%$. The number of feature combinations with better or equal accuracies than the full set of features might be seen as an indicator for a successful feature selection in terms of improved accuracy or reduced feature set sizes. In our experiments, these numbers range from 95.4% ($d_{1}$) to 0.2% ($d_{8}$). Feature subsets with higher accuracies occur more frequently for datasets $d_{1}-d_{5}$ [35.2%; 95.4%]. For datasets $d_{6}-d_{7}$ the accuracy of the full feature set can be achieved by [15.5%; 39.3%] of all feature subsets. Here, the influence of uninformative features can be absorbed in most cases. The lowest number of better or equal feature combinations was observed for dataset $d_{8}$ [0.15%;0.6%]. Here, the full set of features is required for achieving the optimal accuracy. The corresponding fitness landscape is not in favor of feature selection.

It is interesting to see that especially the multivariate feature combinations tend to form multimodal distributions (according to accuracy) indicating the existence of multiple quality classes of feature combinations. These classes are mainly determined by the presence or absence of specific feature combinations, which dominate the influences of the corresponding signature. For larger feature set sizes the different subpopulations are likely to be absorbed by the overall central tendency.

### Cross-validation results

The results of the CV experiments are summarized in Table 4. Here, the classification accuracies in external $10 \times 10$ CV experiments and the number of necessary features for *e*-*k*-NN leading to maximal accuracies are depicted.

In total, 7 out of 8 datasets obtained higher accuracies using *e*-*k*-NN instead of *k*-NN. Here, in 7 of 8 cases *e*-1-NN outperformed 1-NN, in 6 of 8 experiments *e*-3-NN led to higher accuracies than 3-NN, for 6 of 8 datasets *e*-5-NN outperformed 5-NN and in 7 of 8 cases *e*-7-NN achieved a better performance than 7-NN. The predictive performance of *k*-NN can be improved by up to $28.8\%$ using *e*-*k*-NN. On average, an improvement of up to $11.2\%$ (*e*-5-NN) in accuracies can be observed.

The variants of *e*-*k*-NN selected $[14.4\%; \,88.3\%]$ (average: $42.8\%$) of all features. The lowest feature numbers were needed by $d_{3}$ while outperforming the traditional *k*-NN by at least $15.2\%$. $d_{8}$ required the largest feature set, leading to a decline in accuracy by at least 0.4% compared to *k*-NN.

For 5 out of 8 datasets, the overall highest accuracies were achieved by a variant of *e*-*k*-NN. The standard *k*-NN performed best for 1 out of 8 datasets. In 1 out of 8 cases the *L*2-SVM and C4.5 outperformed the other classifiers, respectively. Over all 8 datasets, the *L*1-SVM, CART, LVQ and NCC never achieved the best performance.

**Table 4:** Results of the $10 \times 10$ CV experiments. The table reports the achieved accuracies and in the number of selected features (mean ± stdev. in %). *k*-NN denotes conventional CV simulation, *e*-*k*-NN refers to exhaustive FSS. The highest mean accuracy per dataset is printed in bold.

Classifier	Datasets							
	$d_{1}$	$d_{2}$	$d_{3}$	$d_{4}$	$d_{5}$	$d_{6}$	$d_{7}$	$d_{8}$
**Achieved accuracy in % (mn. ± std.)**								
*e*-1-NN	83.7±1.4	90.5±1.9	84.9±0.5	71.4±2.2	86.0±1.7	**97.0±0.2**	92.0±0.4	80.7±1.1
*e*-3-NN	83.9±0.9	93.3±1.1	**85.9±0.4**	76.6±2.8	84.6±1.8	96.8±0.1	91.5±0.3	80.1±1.0
*e*-5-NN	84.8±0.5	93.0±1.6	85.0±0.9	78.6±2.1	84.6±1.3	96.7±0.1	91.1±0.2	80.6±1.4
*e*-7-NN	**85.1±0.4**	91.0±1.5	84.9±0.8	**79.0±1.2**	**88.1±1.7**	96.3±0.2	90.7±0.4	78.1±1.2
1-NN	54.8±1.5	76.6±0.8	65.8±0.5	59.9±2.6	84.4±1.3	96.5±0.1	90.2±0.1	**81.7±0.9**
3-NN	59.5±1.7	72.4±1.4	67.3±0.5	52.6±2.9	85.0±0.9	95.3±0.3	90.5±0.2	81.2±1.1
5-NN	62.7±1.0	70.8±1.9	68.6±0.4	52.5±1.7	85.0±1.2	94.3±0.2	89.7±0.3	81.0±0.7
7-NN	64.2±0.8	70.6±2.3	69.7±0.5	51.6±2.1	83.9±1.0	93.5±0.2	89.4±0.3	81.3±1.1
*L*1-SVM	54.7±3.8	83.5±3.5	76.7±1.3	58.9±2.9	68.2±5.3	81.2±2.7	80.5±2.9	70.7±1.8
*L*2-SVM	$82.5\pm 0.5$	**95.8±1.0**	85.2±0.2	74.3±1.1	86.5±0.8	96.3±0.1	89.8±0.2	75.5±1.1
CART	81.3±1.6	88.3±0.9	85.6±0.9	66.9±1.9	86.6±1.4	91.9±0.2	86.5±0.1	66.0±1.6
C4.5	81.7±1.5	92.4±1.6	83.9±0.7	73.3±3.5	84.6±1.9	96.8±0.2	**93.0±0.4**	66.3±1.9
LVQ	57.9±2.3	69.8±2.8	64.8±0.9	46.0±4.3	77.6±2.6	73.5±1.0	80.0±1.0	57.7±3.1
NCC	50.1±0.5	72.5±0.6	65.0±0.2	49.2±1.0	72.1±0.5	72.0±0.2	63.8±0.1	45.4±0.7
**Number of selected features in % (mn. ± std.)**								
*e*-1-NN	32.8±1.9	46.8±1.8	14.4±1.4	48.4±1.9	34.2±3.4	32.2±1.4	44.2±1.5	86.8±1.8
*e*-3-NN	38.8±2.5	45.4±2.1	26.5±1.1	59.7±1.7	28.9±3.1	33.8±1.1	36.1±1.9	88.2±1.8
*e*-5-NN	29.8±2.8	46.5±3.0	27.5±0.5	58.6±1.7	28.8±2.0	32.3±0.8	37.1±6.1	86.2±0.7
*e*-7-NN	30.3±2.9	46.3±2.7	26.0±0.8	48.8±1.2	21.2±2.6	32.1±1.3	40.0±6.6	80.7±1.9

## Discussion and conclusion

The design of a suitable feature subset is one of the major tasks in training a classification model with a high impact on its final predictive performance. Adapted to the same task a classification model might be highly predictive or non-predictive if based on different feature representations of a data collection. This effect is not only of interest for modelling purposes but also in a broader scientific context, where informative features might reveal new hypotheses. The second one extends the traditional scope of feature subset selection from higher-dimensional settings to lower-dimensional ones.

In both scenarios, high coverage of all feature combinations would be desirable for detecting optimal solutions, characterizing their neighborhoods, identifying potential alternatives and finally for outlining the landscape of the underlying search space. However, this aim becomes more and more unrealistic for higher dimensions due to the exponential nature of the exponential growth of the number of feature combinations. Here, additional evaluations can enlarge the exploration of the search space. Those aims can be supported by fast evaluation criteria as they allow for higher coverage in the same amount of time. Nevertheless, these evaluation criteria should also be robust to counteract the effects of overfitting. Time-consuming re- or subsampling experiments might be required to ensure this property. Complexity reduction should therefore focus on this aspect.

In this work, we address this scenario by proposing two techniques for efficient cross-validation traversals that improve the runtime of distance-based feature subset selection and even extend the range of exhaustive feature subset evaluations. Both are accompanied by theoretical findings on their computational complexity and error bounds. The first one, a traversal strategy, reduces the computational complexity of multivariate distance calculations to the complexity of univariate ones. Dimensionality therefore no longer affects the generation of distance matrices, if feature selection experiments are performed incrementally as for example in forward-selection or backward-elimination wrappers. We show the potential of these traversals in exhaustive feature selection experiments, which comprise an exponential number of distance evaluations. For these enumerations, we provide an optimal walkthrough that not only allows the use of univariate distance calculations for each feature signature but also the use of arbitrary splits for load-balanced parallelization. In our experiments, the dimensionality-invariant techniques allowed for the evaluation of $2^{40}-1$ marker combinations. This number is independent of the dimensionality of the feature subsets. It is clearly out of range for de novo calculations, which become even more complex for higher dimensions.

The second approach addresses the computational complexity of cross-validation experiments of distance-based classifiers. This type of experiment is mainly designed for simulations of independent training and test runs and therefore assumes a de novo calculation of classification models and predictions. Nevertheless, memoization techniques might be used to improve training and evaluation time. For the *k*-Nearest Neighbor classifier, memoization can be applied twice. First, the repetitive calculation of distances between training and test samples can be replaced by providing a global distance matrix as lookup structure. Second, the global *k* nearest neighbors extracted from this matrix are likely to correspond to the local *k* nearest neighbors of an individual experiment, which allows for a probabilistic retrieval.

The memoization of *k* nearest neighbors reveals an inherent relation of the leave-one-out (global structure) and other cross-validations (local structures), which can not only be applied for runtime improvement but also for error estimation. We provide a theoretical error bound on the general cross-validation error based on the error of the leave-one-out. It might be used for replacing standard cross-validation by leave-one-out experiments if upper bounds are sufficient.

Our experiments did not only allow the comparison of runtimes but also the exploration of exhaustive feature selection experiments. Although typically omitted due to their exponential computational complexity exhaustive feature selection experiments have some interesting theoretical properties. In contrast to (standard) heuristic or stochastic feature selection strategies, exhaustive feature selection -per definition- does not suffer from subsampling effects. Global optimal solutions can not be missed. It therefore allows to provide a direct relation between the chosen optimization criterion and the analyzed data. Although other optimization techniques also allow to provide (global) optimal solutions for specific criteria, the exhaustive one is the only general purpose optimization technique with this property. It is therefore of interest for a wider range of applications.

Exhaustive feature selection achieves this property by conducting a census on all feature combinations. The corresponding data can afterwards be used for providing a fitness landscape, which outlines feature interactions and their influence on a classifier’s performance. For the *k* Nearest Neighbor classifier, we observed a beneficial influence of predictive standalone markers. They are a major ingredient of the top-scoring features combinations. The more features a signature reassembles the higher gets its similarity to the full signature. This not only affects the similarity of the corresponding distance matrices but also the similarity of the induced accuracies. As the *k* Nearest Neighbor classifier is unable to conduct (internal) feature-wise decisions its performance is influenced by all components of the underlying feature signature. Although we observe synergistic effects especially for smaller feature signatures, larger ones are likely to be overshadowed by uninformative components leading to a decline of accuracy. Individual features can only dominate the performance of the overall classifier if they outperformed the remaining features in scale. This effect might be different for other types of classifiers. Therefore, our future work will focus on the generation of fitness landscapes for other classification models, e.g., the *L*2-SVM and NCC. While our scheme can be transferred to other distance-based classifiers, exhaustive screens might not as easily be conducted for other types of models. Other acceleration techniques will be required for these model classes.

## Supplementary Information

Supplementary Information.

## Data Availability

All data generated or analysed during this study are included in this published article and its supplementary information file. Artificial data was generated by random sampling from a uniform distribution (see “Runtime experiments” section). The real-world data that was analysed is publicly available from the UCI machine learning repository https://archive.ics.uci.edu.
