# Supplementary material for: Efficient cross-validation traversals in feature subset selection
Source: Sci Rep. 2022 Dec 12;12:21485. doi: 10.1038/s41598-022-25942-4 (PMC9744898; doi:10.1038/s41598-022-25942-4)
Supplement: Supplementary file 1 — Supplementary Information. [file 41598_2022_25942_MOESM1_ESM.pdf]

Efficient cross-validation traversals in feature subset selection

ABSTRACT

Supplementary simulations.

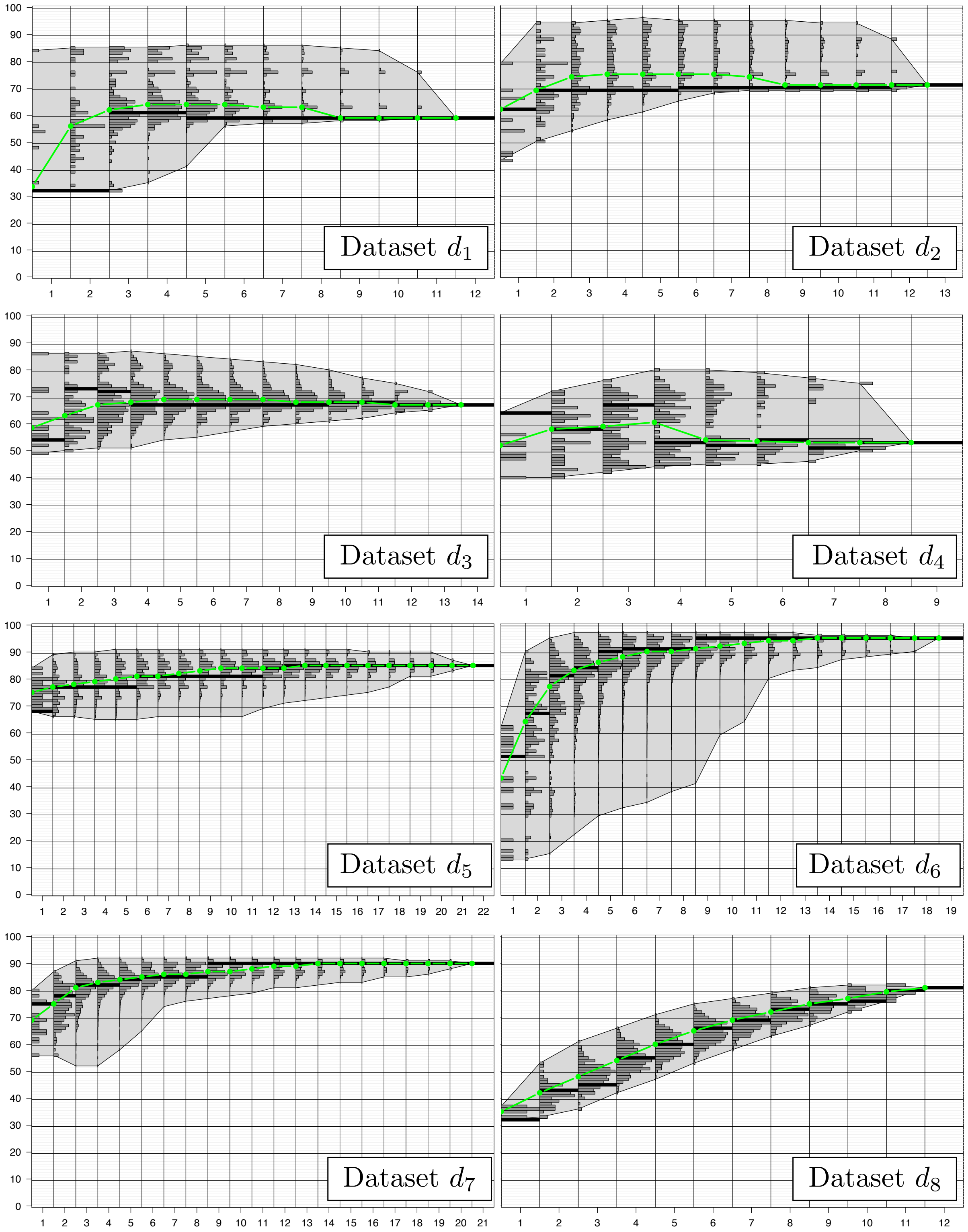

Figure S1. **Fitness landscapes**. The figure shows the fitness landscapes of e-k-NN, $k = 3$, for datasets $d_1 - d_8$. A fitness landscape provides the accuracies of all analyzed feature combinations. They are organized in histograms for the individual feature set sizes $\hat{n}$ (column-wise). The height of a histogram is normalized by the mode of the corresponding set. The underlying polygon combines the minimal and the maximal accuracies achieved for $\hat{n}$. For each feature combination, the mode is shown in black within the single histograms. The median of each combination is depicted in green.

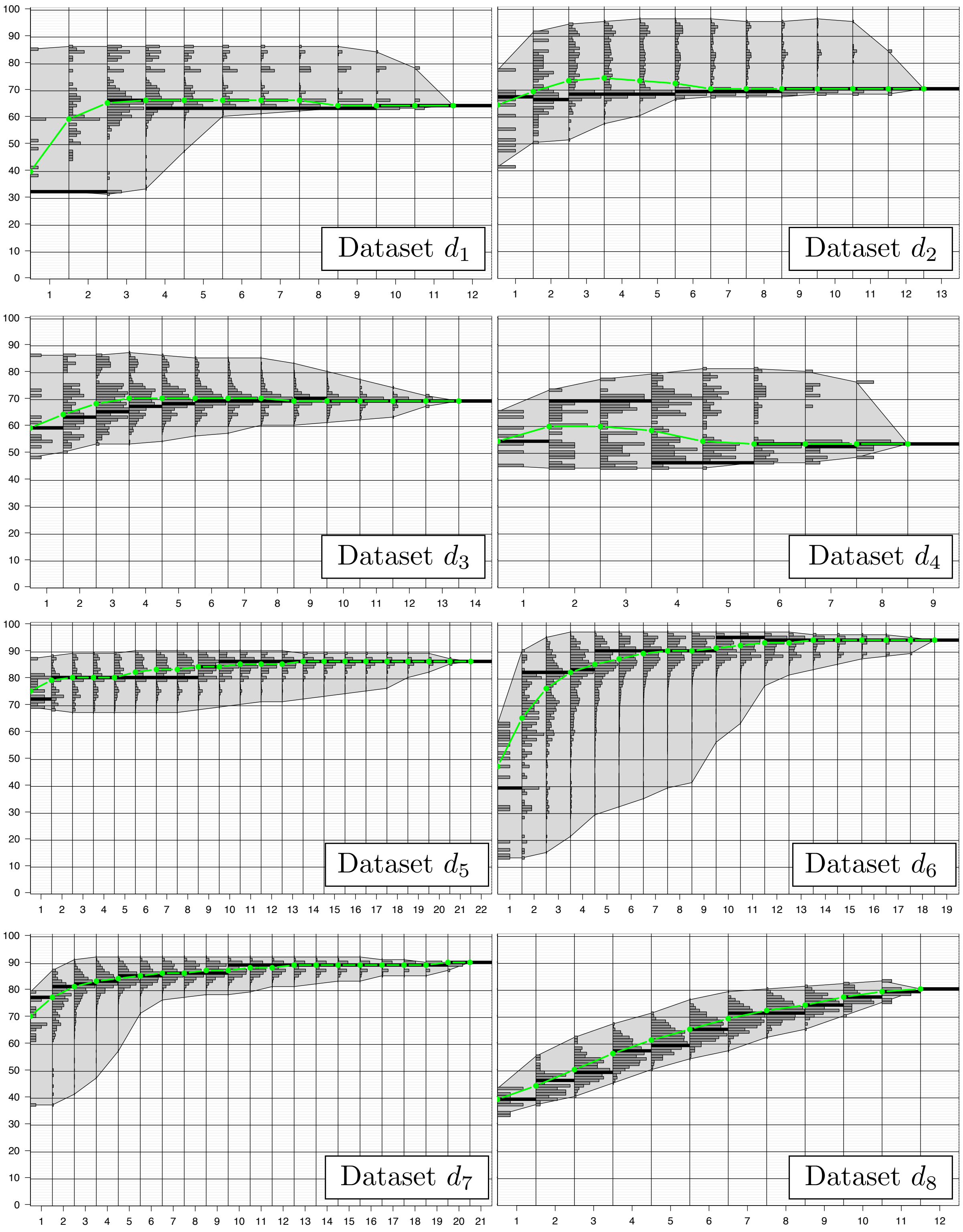

Figure S2. Fitness landscapes. The figure shows the fitness landscapes of e-k-NN, $k = 5$, for datasets $d_1 - d_8$. A fitness landscape provides the accuracies of all analyzed feature combinations. They are organized in histograms for the individual feature set sizes $\hat{n}$ (column-wise). The height of a histogram is normalized by the mode of the corresponding set. The underlying polygon combines the minimal and the maximal accuracies achieved for $\hat{n}$. For each feature combination, the mode is shown in black within the single histograms. The median of each combination is depicted in green.

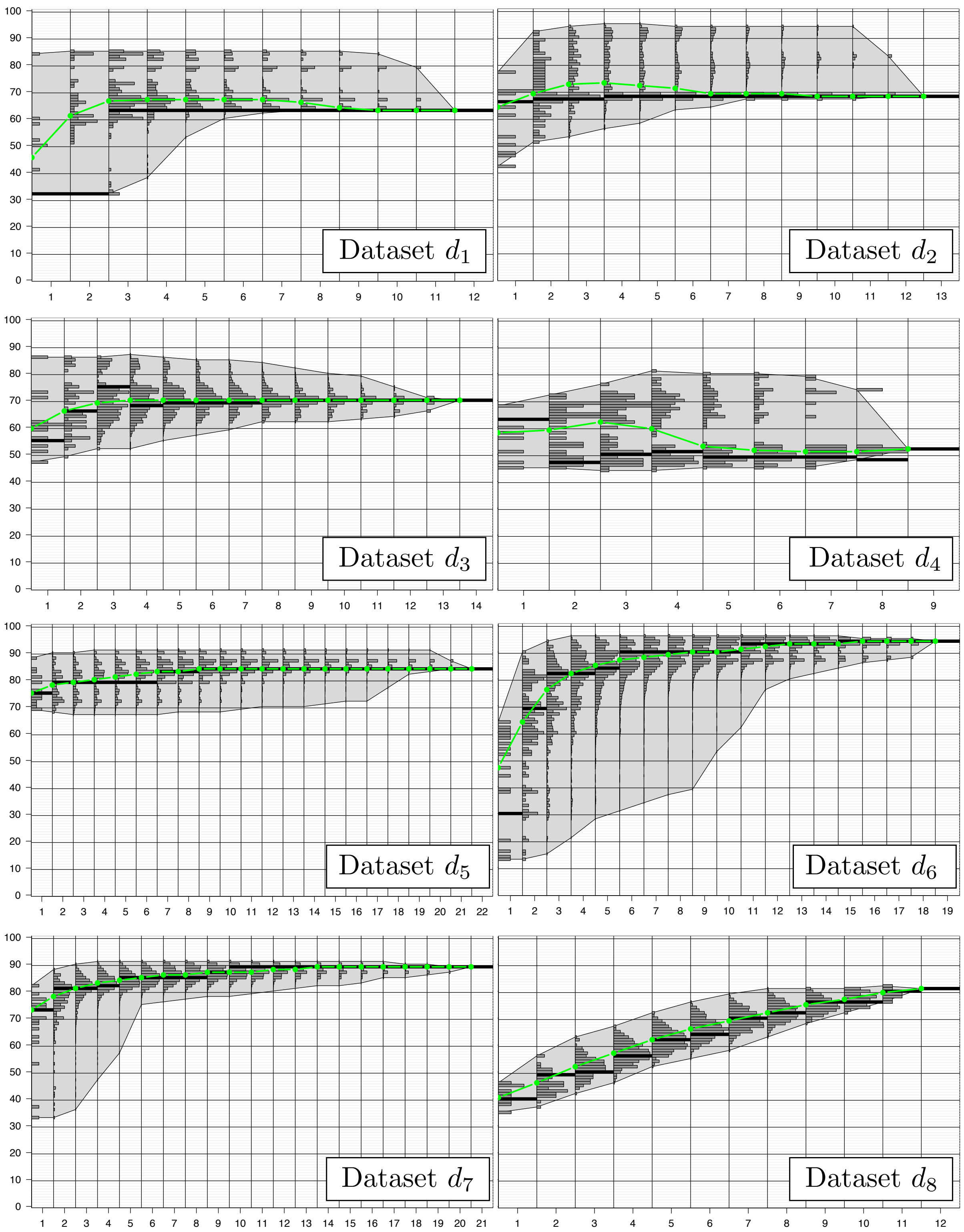

Figure S3. Fitness landscapes. The figure shows the fitness landscapes of e-k-NN, $k = 7$, for datasets $d_1 - d_8$. A fitness landscape provides the accuracies of all analyzed feature combinations. They are organized in histograms for the individual feature set sizes $\hat{n}$ (column-wise). The height of a histogram is normalized by the mode of the corresponding set. The underlying polygon combines the minimal and the maximal accuracies achieved for $\hat{n}$. For each feature combination, the mode is shown in black within the single histograms. The median of each combination is depicted in green.
